# Complete Microbiota Engraftment Is Not Essential for Recovery from Recurrent *Clostridium difficile* Infection following Fecal Microbiota Transplantation

**DOI:** 10.1128/mBio.01965-16

**Published:** 2016-12-20

**Authors:** Christopher Staley, Colleen R. Kelly, Lawrence J. Brandt, Alexander Khoruts, Michael J. Sadowsky

**Affiliations:** a BioTechnology Institute, University of Minnesota, St. Paul, Minnesota, USA; b Department of Medicine, The Warren Alpert Medical School of Brown University, Providence, Rhode Island, USA; c Women’s Medicine Collaborative, The Miriam Hospital, Providence, Rhode Island, USA; d Department of Medicine, Division of Gastroenterology, Albert Einstein College of Medicine, Montefiore Medical Center, Bronx, New York, USA; e Division of Gastroenterology and Center for Immunology, Department of Medicine, University of Minnesota, Minneapolis, Minnesota, USA; f Department of Soil, Water, and Climate, University of Minnesota, St. Paul, Minnesota, USA

## Abstract

Bacterial communities from subjects treated for recurrent *Clostridium difficile* infection (rCDI) by fecal microbiota transplantation (FMT), using either heterologous donor stool samples or autologous stool samples, were characterized by Illumina next-generation sequencing. As previously reported, the success of heterologous FMT (90%) was superior to that of autologous FMT (43%) (*P* = 0.019), and post-FMT intestinal bacterial communities differed significantly between treatment arms (*P* < 0.001). Subjects cured by autologous FMT typically had greater abundances of the *Clostridium* XIVa clade and *Holdemania* bacteria prior to treatment, and the relative abundances of these groups increased significantly after FMT compared to heterologous FMT and pre-FMT samples. The typical shift to post-FMT, donor-like assemblages, featuring high relative abundances of genera within the *Bacteroidetes* and *Firmicutes* phyla, was not observed in the autologous FMT subjects. Autologous FMT patient bacterial communities were significantly different in composition than those for heterologous FMT patients and donors (*P* < 0.001). The SourceTracker program, which employs a Bayesian algorithm to determine source contributions to sink communities, showed that patients initially treated by heterologous FMT had significantly higher percentages of engraftment (i.e., similarity to donor communities, mean value of 74%) compared to those who suffered recurrence following autologous FMT (1%) (*P* ≤ 0.013). The findings of this study suggest that complete donor engraftment may be not necessary if functionally critical taxa are present in subjects following antibiotic therapy.

## IMPORTANCE

This study provides a detailed characterization of fecal bacterial communities in subjects who participated in a previously published randomized clinical trial to treat recurrent *C. difficile* infection (rCDI). Bacterial communities were characterized to determine differences between subjects who received fecal bacteria either from healthy donor stool samples or their own stool samples as “placebo” in order to determine which groups of bacteria were most important in achieving a cure. The results of this study suggested that bacteria associated with secondary bile acid metabolism could potentially provide resistance to infection and that complete transfer of healthy donor microorganisms was not necessary to resolve CDI following unsuccessful antibiotic treatment.

## INTRODUCTION

The endogenous intestinal microbial community is comprised of commensal species, primarily bacteria within the phyla *Firmicutes* and *Bacteroidetes*, that play a critical role in human health by facilitating nutrient metabolism and protecting against pathogen colonization (1). However, disruption of the endogenous community, typically following administration of antibiotics (2, 3), allows for the proliferation of enteric pathogens, including *Clostridium difficile* (4). Over the last several decades, the morbidity, severity, and fatality statistics for *C. difficile* infection (CDI) have been on the rise (5–7), and this has been attributed, in part, to the emergence of hypervirulent *C. difficile* strains with the NAP1/BI/ribotype 027 genotype. These hypervirulent clostridia show greater resistance to antibiotics and increased toxin production (8, 9).

Standard treatment for CDI involves orally administered metronidazole or vancomycin, but these treatments have been shown to result in recurrence in 27% and 24% of subjects, respectively (10). Recurrent CDI (rCDI) typically occurs within 4 weeks (6), resulting either from reinfection by a new strain or persistence of spores from the initial infecting strain (11). The risk of further rCDI increases with each subsequent recurrence: 30% after the first recurrence and up to 60% following two recurrences (6, 12).

Since the proliferation of *C. difficile* typically occurs following a reduction in diversity of the intestinal microbial community, restoration of this diversity provides a promising avenue to treat rCDI (13, 14). Members of the intestinal microbial community have previously been shown to effectively impair, or prevent, growth of *C. difficile in vitro* (15), and a recent study has specifically identified several species, including *Clostridium scindens*, that may play a primary role in resistance to *C. difficile* infection by production of secondary bile acids (16).

Fecal microbiota transplantation (FMT), first described in 1958 (17), involves transplantation of healthy donor stool with its constituent microorganisms into subjects with various diseases, e.g., rCDI, to restore intestinal microbial diversity and function. FMT has received increasing attention recently as an effective treatment for rCDI (18–24) with cure rates of >90% (19, 20, 22, 24). Furthermore, several clinical trials have demonstrated the efficacy of FMT over vancomycin alone (25), as well as the equality of frozen and fresh donor material (26) to cure rCDI. Engraftment of host microbiota has been demonstrated at 3 days post-FMT (21). Intestinal microbial communities in subjects’ feces were significantly correlated with those in donors (23), and the new community assemblage was stable for 4 months post-FMT (22). However, a study in mice and humans found that restoration of donor-like beta diversity, i.e., expansion of *Firmicutes* and *Bacteroidetes*, did not always correspond to direct restoration of an individual’s alpha diversity (species richness and evenness), suggesting that specific species may be necessary for resistance to *C. difficile* (16).

Changes in the composition of the intestinal microbial community as a result of antibiotic exposure in patients with rCDI have been well characterized by using several molecular approaches, including next-generation sequencing (13, 16, 21–23). In nearly every case, microbial community alpha diversity decreases significantly compared to that of healthy individuals, with a drastic reduction in the members of the typically dominant *Firmicutes* and *Bacteroidetes* phyla and an expansion of *Proteobacteria*, especially members of the *Enterobacteriaceae* family (21–23). After FMT, the patient’s intestinal microbiome, in contrast, shows a reduction in *Proteobacteria* and an expansion of the families *Ruminococcaceae*, *Lachnospiraceae*, and *Clostridiaceae* in the phylum *Firmicutes*, as well as the families *Bacteroidaceae*, *Rikenellaceae*, and *Porphyromonadaceae* in the phylum *Bacteroidetes* (21, 22).

The dysbiosis associated with rCDI and the subsequent post-FMT shifts in community composition have been broadly described taxonomically. Furthermore, communities of healthy individuals are known to show plasticity within a certain dynamic range (23). Therefore, while several species may be identified as potentially conferring resistance to rCDI (16), it remains unclear to what extent these specific members are critically essential for recovery. This finding, however, does suggest the possibility that certain pre-FMT assemblages may impact the success and/or necessity of FMT after selective antibiotic pressures are removed. Moreover, a randomized double-blind clinical trial in which subjects were treated for rCDI by heterologous (donor stool) FMT (H-FMT) or autologous (the patient’s stool) FMT (A-FMT) as “placebo” revealed that, while heterologous FMT resulted in significantly higher rates of cure than autologous FMT (90% versus 43%; *P* = 0.019), autologous FMT was, in some cases, successful (27). Notably, bacterial communities in feces from subjects who were cured by autologous FMT were significantly differentiated from those of subjects treated using heterologous FMT, initially or as a follow-up rescue therapy, although these differences were not explored in detail (27).

In the current study, the microbiomes of subjects enrolled in the randomized FMT clinical trial at the Rhode Island site (27) were extensively characterized using Illumina next-generation sequencing of the 16S rRNA gene. The patient cohort at the New York site had significantly greater duration of CDI prior to treatment and significantly different pre-FMT assemblages, and nearly all the patients recovered following autologous FMT (27); thus, they were excluded from the analysis presented here to reduce confounding variables. We hypothesized that unique taxa would be present in pre-FMT samples from subjects cured by autologous FMT that were absent from those who relapsed. It was further hypothesized that samples from patients treated by heterologous FMT would show more rapid normalization of their microbiome compared to autologous FMT recipients. Subjects who relapsed after autologous FMT were treated with a follow-up FMT using donor stool (F/U-FMT), and the microbiomes of subjects treated with follow-up FMT were hypothesized to closely resemble those of heterologous FMT subjects. Finally, the extent of engraftment (i.e., transfer of taxa from donors to patients) of donor communities in subjects was evaluated for the following two aims: (i) to assess the utility of the SourceTracker software program (28) that employs a Bayesian algorithm to measure engraftment, defined as the percentage similarity in patient (sink) fecal communities compared with those from donors (source); and (ii) to determine how the success of engraftment varied based on treatment. Results of this study provide greater insight into the potential impacts of pre-FMT assemblages on the efficacy of FMT and further describe shifts in the microbiome associated with cure in the absence of donor material. In addition, SourceTracker is shown to be a useful tool to determine the extent and stability of donor engraftment.

## RESULTS

### Alpha diversity and community composition of donor and patient fecal samples.

Among all samples, using 25,000 normalized reads per sample, a mean estimated Good’s coverage of 99.5% ± 0.2% was achieved with the Illumina sequencing platform. Alpha diversity, as measured by both the Shannon index (richness and evenness) and abundance-based coverage estimate (ACE) metric (species richness), differed significantly among samples (*P* < 0.0001) (Table 1). Donor samples and samples from subjects treated by heterologous FMT had significantly greater alpha diversity (27), based on both parameters, than did pre-FMT samples and samples from subjects who failed to achieve CDI cure after autologous FMT. Neither Shannon nor ACE indices differed among samples from subjects who were cured by autologous FMT and those treated with heterologous FMT (*P* ≥ 0.075).

**TABLE 1:** Alpha diversity indices of donor and patient samples treated

Sample or clinical outcome and treatment arm^a^	Time point (wks)	*n*	Coverage	Diversity index^b^	
				Shannon	ACE
Pre-FMT samples		13	99.7 ± 0.1	2.42 ± 0.53 AB	305 ± 97 A
Donor samples		9	99.4 ± 0.2	3.47 ± 0.49 C	787 ± 296 B
Cure					
H-FMT	2	7	99.5 ± 0.1	3.40 ± 0.67 C	678 ± 123 B
	8	7	99.4 ± 0.1	3.55 ± 0.39 C	829 ± 213 B
H-FMT follow-up	2	9	99.4 ± 0.2	3.52 ± 0.53 C	790 ± 306 B
	8	7	99.3 ± 0.2	3.68 ± 0.43 C	966 ± 332 B
A-FMT	2	3	99.6 ± 0.1	3.21 ± 0.36 BC	531 ± 143 AB
	8	4	99.6 ± 0.1	3.06 ± 0.32 ABC	521 ± 93 AB
Failure and A-FMT	2	2	99.7 ± 0.0	1.63 ± 0.25 A	299 ± 28 A
	8	1	99.6	2.47 ABC	435 AB
*P* value^c^				<0.0001	<0.0001

a H-FMT, heterologous FMT; A-FMT, autologous FMT.

b Values are means ± standard deviations. Values within a column with the same letter did not vary by Tukey’s posthoc test at α = 0.05.

c *P* values reflect the value of the Fisher’s F test of the ANOVA model, treating each entry in the column as a separate group. Separate models were calculated for Shannon and ACE indices.

**FIG 1:**
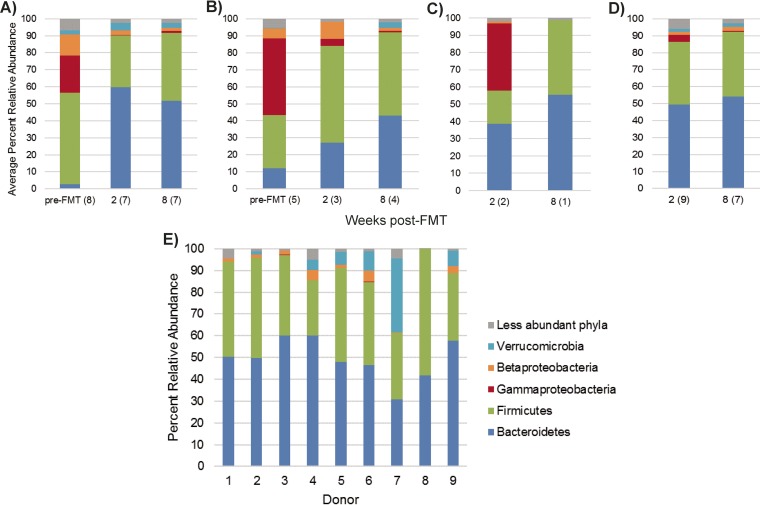
Distribution of phyla in patient and donor fecal samples. (A to D) Distribution of phyla in fecal samples from patients cured by heterologous FMT (A) or autologous FMT (B) and patients who had recurrence following autologous FMT (C) and received a follow-up heterologous FMT (D). (E) Distribution of phyla in samples from individual donors. The numbers within parentheses reflect sample sizes, and phylum percentages were averaged among patient samples.

Generally, when classified to phyla, pre-FMT patient samples showed greatly reduced relative abundances of members of the phylum *Bacteroidetes* and an expansion of the phylum *Proteobacteria*, predominantly *Gamma*- and *Betaproteobacteria*, relative to donor fecal samples (Fig. 1), as observed previously (27). Treatment by heterologous FMT (initially or after follow-up FMT) resulted in a significant increase of *Bacteroidetes* (*P* < 0.001) by the 2-week time point that did not differ from donor proportions (*P* = 0.990), but this increase was not significant in patients who received autologous FMT (*P* = 0.354). The microbiomes of subjects treated by autologous FMT showed a slower taxonomic shift toward a donor-like assemblage, and the relative abundances of phyla did not differ significantly (*P* ≥ 0.137) between autologous FMT recipients at 8 weeks post-FMT and donors, for the taxa shown in Fig. 1.

Among abundant genera (present at a mean of >1.0% of sequence reads among all samples), several significant shifts were observed among treatment groups (heterologous FMT, autologous FMT, and follow-up FMT; see Fig. S1 and S2 in the supplemental material). The abundances of *Klebsiella* were significantly lower at 2 weeks post-FMT among all groups (*P* ≤ 0.003). Similarly, the relative abundances of *Bacteroides* were significantly greater among all treatment groups at the 8-week time point than pre-FMT (*P* ≤ 0.012). However, the abundances of *Parabacteroides* were significantly greater (*P* ≤ 0.030) only among heterologous FMT and follow-up FMT patients relative to pre-FMT patients at the 2-week time point and did not increase significantly among autologous FMT patients (*P* ≥ 0.998). Heterologous FMT, but not follow-up FMT samples, showed a greater relative abundance of *Gemmiger* (within the family *Ruminococcaceae* of the *Firmicutes*; *P* = 0.044) relative to autologous FMT when all time points were considered together. Furthermore, follow-up FMT samples had greater abundances of *Alistipes* relative to heterologous FMT communities (*P* = 0.012), but they did not differ from autologous FMT samples, considering all time points.

### Relationship of the pre-FMT community to the clinical outcome.

**FIG 2:**
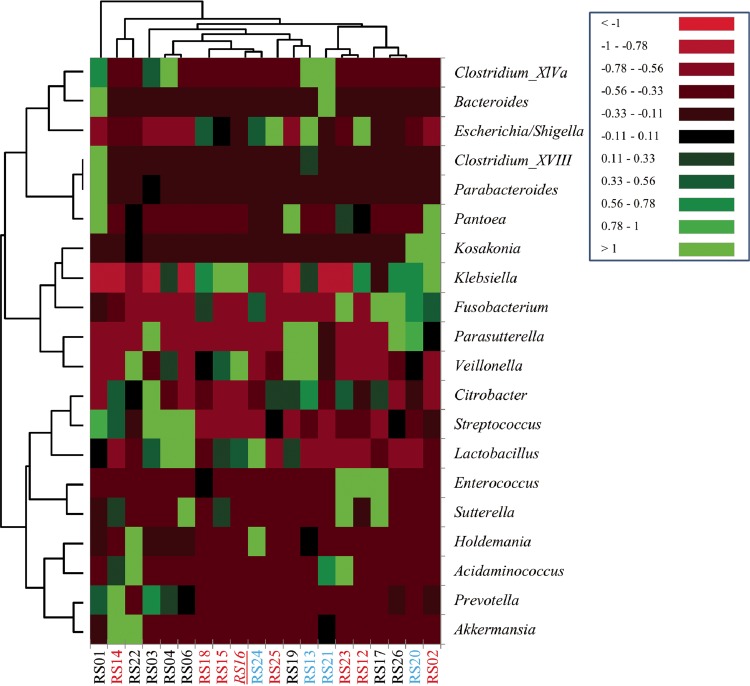
Heat map of abundant genera (mean of >1.0% of sequence reads) in pre-FMT samples from Rhode Island subjects (RS01 to RS26). Samples in red were clinical failures, and the single heterologous FMT failure (RS16) is italicized and underlined. Samples from patients shown in black (receiving heterologous FMT) or blue (receiving autologous FMT) were cured. Dendrograms were calculated using ascendant hierarchical clustering based on Euclidean distances. Scaling was calculated based on minimum (−1) and maximum (+1) percentages.

Evaluation of the distributions of abundant genera revealed that multiple pre-FMT assemblages occurred among subjects treated by heterologous FMT and autologous FMT (Fig. 2). Among the predominant features of pre-FMT communities were high relative abundances of members of the genera *Lactobacillus*, *Klebsiella*, and *Escherichia* or *Shigella*. Comparison of operational taxonomic units (OTUs) revealed that only one OTU, classified as a *Lactobacillus* sp., was common to all samples. Differences in beta diversity, evaluated by analysis of similarity (ANOSIM), were not different between pre-FMT communities when samples were grouped by treatment arm and clinical outcome (*P* = 0.121). However, among autologous FMT patient samples, patients who were cured had greater relative abundances of *Parasutterella* (*P* = 0.030; Fig. 2), although no other differences in genera were significant.

### Shifts in community composition following autologous FMT.

**FIG 3:**
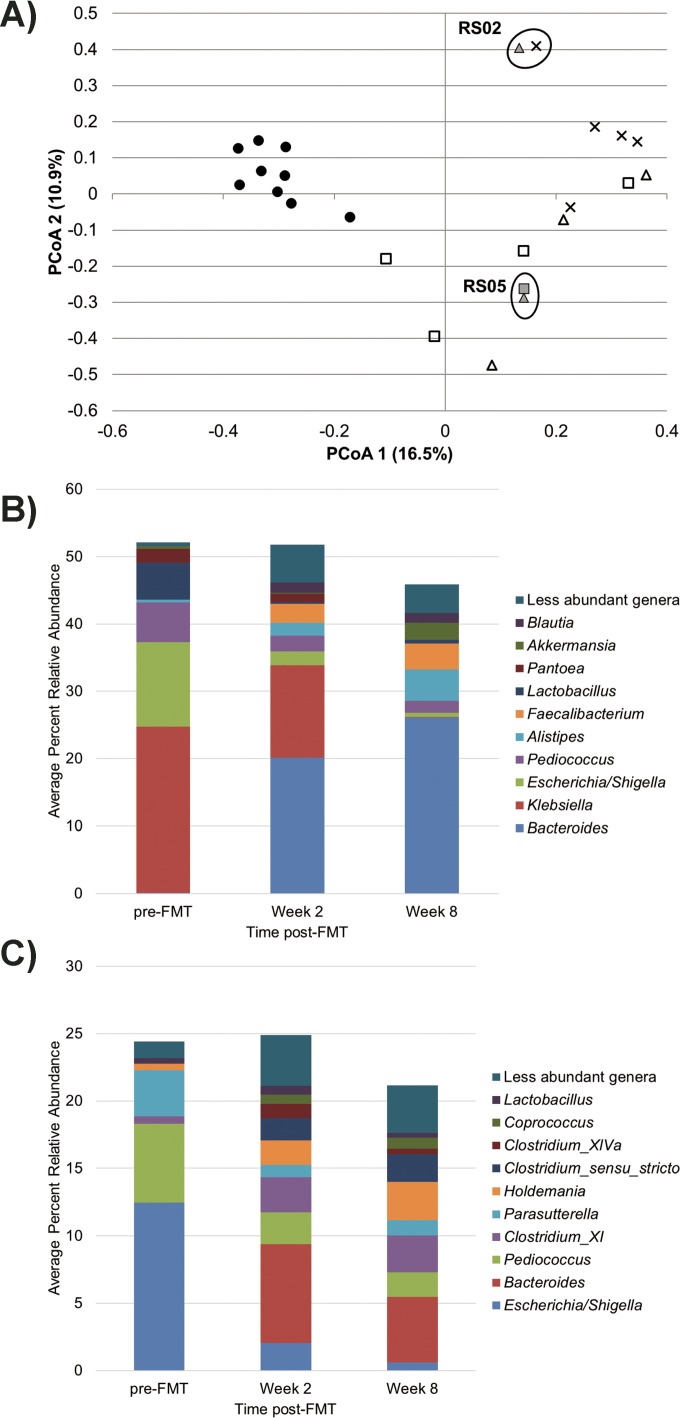
Principal-coordinate analysis and distribution of families influencing ordination position. (A) PCoA (*r*^2^ = 0.45) of autologous FMT subject and donor samples. Symbols: ●, donors; ×, pre-FMT; ▲, 2-week sample; ■, 8-week samples; open symbols, cured subjects; gray symbols, patients who relapsed. Circles indicate samples from subjects who experienced recurrence. The Rhode Island subject number (RS) is given. (B) Family-level classification of OTUs that influenced position along the first PCoA axis (PCoA 1) (as determined by Spearman correlation). (C) Family-level classification of OTUs that influenced position along the second PCoA axis (PCoA 2) (as determined by Spearman correlation).

Samples from subjects initially treated by autologous FMT showed variable trends in higher-resolution (genus-level) taxonomic shifts throughout the study (see Fig. S1 in the supplemental material). Ordination of samples by principal-coordinate analysis (PCoA) (Fig. 3A) revealed that time post-FMT was negatively correlated with sample position along both the *x* and *y* axes (Spearman’s *r* = −0.510 and −0.624 and *P* = 0.055 and 0.015, respectively). OTUs that significantly (*P* < 0.05) affected position along the *x* axis were classified as members of the genera *Bacteroides* and *Alistipes*, genera *Faecalibacterium*, *Blautia*, and *Roseburia,* and genus *Akkermansia* within the phyla *Bacteroidetes*, *Firmicutes*, and *Verrucomicrobia*, respectively (Fig. 3B), and abundances of these taxa were inversely associated with *x*-axis position. Similarly, OTUs significantly (*P* < 0.05) associated with the *y* axis were classified within the genera *Bacteroides* and *Holdemania* and clades of clostridia, the abundances of which also increased inversely to *y*-axis position (Fig. 3C). Notably, *Escherichia* was significantly (*P* < 0.05) positively associated with *y*-axis position.

The bacterial community composition (beta diversity) in autologous FMT samples differed significantly from that of healthy donors and patients receiving heterologous FMT at all time points (*P* < 0.001 by ANOSIM). Among subjects cured by autologous FMT, differences in beta diversity, assessed by ANOSIM, were significant at the 2-week (*P* = 0.024) and 8-week (*P* = 0.010) time points compared to pre-FMT samples. Similarly, among the two patient samples who had recurrences of CDI, differences in beta diversity differed significantly from all pre-FMT samples (*P* = 0.042). Community composition did not vary significantly between the 2- and 8-week time points (*P* ≥ 0.735), regardless of clinical outcome.

Evaluation of autologous FMT samples using SourceTracker, with pre-FMT samples designated the source, revealed a high degree of diversification in community composition following cessation of antibiotic therapy and FMT, and these samples showed very low levels of similarity to pre-FMT communities (mean, 1.1% ± 1.8%; *n* = 10). One patient, however, Rhode Island subject RS02, had a bacterial community at 2 weeks that was 14% similar to the original, pre-FMT community, while all other patients showed ≤5% similarity, and this patient experienced recurrence following this time point.

### Comparison of heterologous FMT and follow-up FMT community compositions.

**FIG 4:**
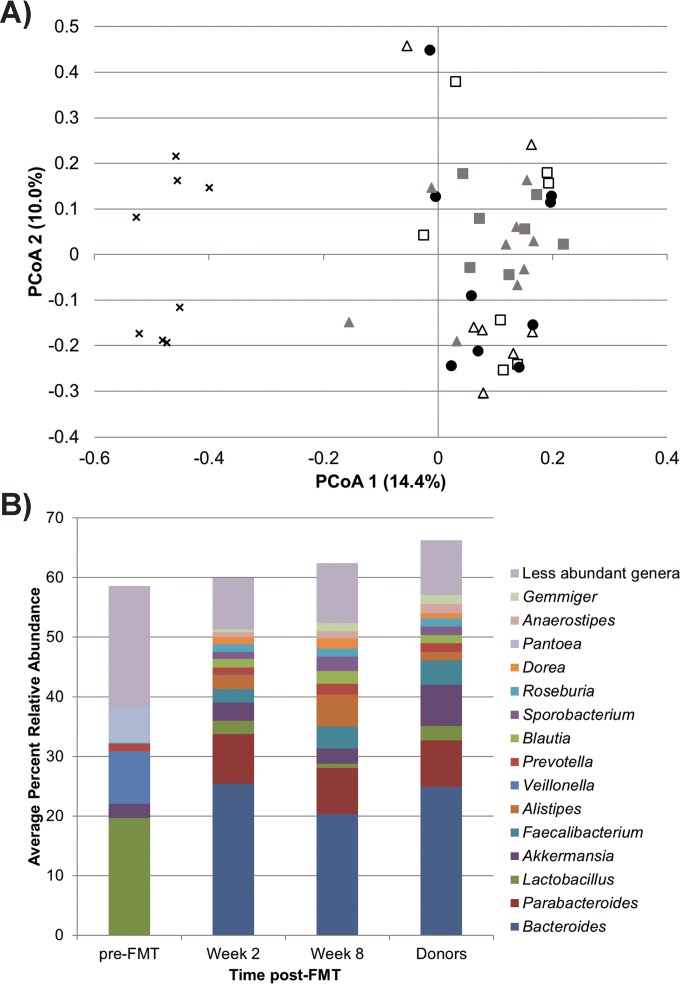
Principal-coordinate analysis and distribution of families influencing ordination position. (A) PCoA (*r*^2^ = 0.62) of subjects treated by heterologous FMT (H-FMT) or follow-up FMT (F/U-FMT) and donor samples. Symbols: ●, donors; ×, pre-FMT; ▲, 2-week samples; ■, 8-week samples; open symbols, H-FMT; gray symbols, F/U-FMT. (B) Family-level classification of OTUs that influenced position along PCoA 1, as determined by Spearman correlation.

The community compositions of post-FMT samples from subjects receiving either heterologous FMT or follow-up FMT differed significantly from pre-FMT samples (*P* < 0.001) (Fig. 4). At either 2- or 8-week time points, beta diversity differences between heterologous FMT and follow-up FMT communities did not differ significantly (*P* = 0.399 and 0.810). Community compositions in these samples also did not differ significantly from those in healthy donor samples (*P* ≥ 0.173).

The microbiomes of subjects treated by heterologous FMT generally underwent stepwise shifts at the 2-week and 8-week time points, as the relative abundance of dominant genera approached that of donor samples (see Fig. S2 in the supplemental material). While trends appeared to be donor specific, a reduction in the *Bacteroides* genus was generally observed. However, increases of other genera within the *Bacteroidetes* and *Firmicutes* phyla were significantly related to ordination position along the *x* axis (Fig. 4), suggesting diversification within these phyla in subjects receiving either heterologous FMT or follow-up FMT.

Bacterial communities in post-FMT samples receiving either randomized or follow-up donor material had a mean community similarity of 74.4% ± 19.7% to the bacterial communities of donors. However, there were variable amounts of engraftment, determined as a percentage of the donor community in patient samples, ranging from approximately 30% to nearly 100% similarity at the 2-week time point (see Fig. S3 in the supplemental material). Characterization of engraftment at the 8-week time point revealed no consistent trends as to whether donor similarity increased or decreased between the 2- and 8-week time points (Fig. S3). While some subjects showed a reduction in donor microbial community similarity, others showed an increase in similarity. Engraftment percentage differences did not vary significantly between time points (*P* = 0.613). However, subjects who initially received heterologous FMT had a significantly higher percentage of engraftment at 2 weeks (88.7% ± 12.3%) relative to those who received follow-up FMT (60.2% ± 18.1%) (*P* = 0.003), and this was also the case at 8 weeks (87.9% ± 8.6% versus 65.0% ± 19.0%; *P* = 0.013).

### Differentiation of community structure by FMT material.

**FIG 5:**
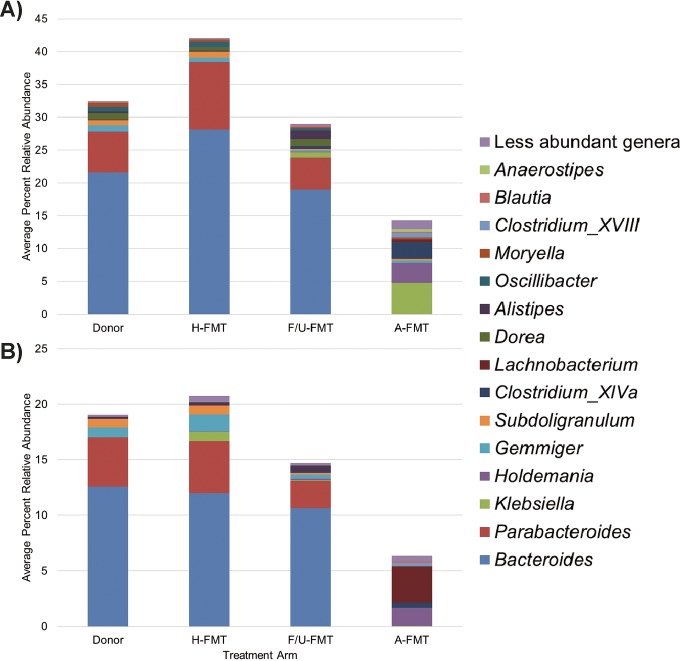
Genus-level classification of OTUs that differed significantly between treatment arms. Differences were evaluated by Kruskal-Wallis test at α = 0.05. (A) Two weeks post-FMT and (B) 8 weeks post-FMT. H-FMT, heterologous FMT; F/U-FMT, follow-up heterologous FMT; A-FMT, autologous FMT.

The amount of community variation (percentage of the community represented by OTUs that showed differing abundance) between donor samples and samples from subjects treated by heterologous FMT, follow-up FMT, and autologous FMT, determined by Kruskal-Wallis test, declined between the 2-week and 8-week time points (*P* < 0.0001) (Fig. 5), indicating an increase in similarity among treatment groups. At 2 weeks post-FMT, OTUs that differed significantly among subjects treated by different FMT procedures accounted for significantly greater percentages of the communities in donor samples and heterologous FMT patient samples than autologous FMT patient samples (*P* ≤ 0.021). The communities in donor, heterologous FMT, and follow-up FMT patient 2-week samples had significantly (*P* < 0.05) greater relative abundances of *Bacteroides* and *Parabacteroides* compared to autologous FMT samples, and autologous FMT samples showed significantly greater relative abundances of *Klebsiella*, *Holdemania*, and *Clostridium* clade XIVa species. Furthermore, autologous FMT samples at 2 weeks post-FMT showed significant and independent clustering from other sample groups by analysis of molecular variance (AMOVA) (*P* < 0.001 by AMOVA; see Fig. S4A in the supplemental material).

At 8 weeks post-FMT, differences in the composition of communities comprised of variable OTUs did not differ significantly among groups (*P* = 0.104). Similar to 2-week samples, samples from donors and from patients treated with heterologous FMT and follow-up FMT were still comprised of significantly greater relative abundances of *Bacteroides* and *Parabacteroides* (Fig. 5). However, autologous FMT samples also showed greater relative abundance of *Parabacteroides* than 2-week samples, suggesting differences in the species within this genus in the autologous FMT and heterologous FMT groups. Furthermore, autologous FMT samples maintained a significantly greater relative abundance of *Holdemania* than other groups. While ordination of samples revealed more similar community composition of 8-week autologous FMT samples to donor, heterologous FMT, and follow-up FMT samples (see Fig. S4B in the supplemental material), independent clustering of autologous FMT samples from others was still significant (*P* = 0.001 by AMOVA).

## DISCUSSION

The importance of specific species in conferring resistance to infection and the therapeutic use of defined consortia of bacteria have begun receiving increased attention as the mechanisms of success of FMT are elucidated (16, 29, 30). A previous study indicated that several species, notably *Clostridium scindens*, may play critical roles in maintaining resistance to CDI (16). These authors indicated that the restoration of secondary bile acid metabolism by this species inhibited *C. difficile* (16). Similarly, FMT has been previously shown to restore bile acid metabolism in subjects with rCDI, who are deficient in secondary bile acid production (30), and fecal concentrations of secondary bile acids have been shown to inhibit germination and vegetative growth of *C. difficile* (31). Taken together, these studies suggest that specific taxa, potentially present in the pre-FMT consortia of subjects, play crucial roles in resistance to CDI.

In this study, we evaluated how variation in pre-FMT communities and FMT done using autologous or heterologous fecal microbiota may relate to clinical outcome in the treatment of rCDI. Subjects cured by autologous FMT typically harbored greater pre-FMT relative abundances of members of the *Clostridium* XIVa clade or *Holdemania* in the family *Erysipelotrichaceae*, as well as *Parasutterella*. As previously suggested (16), subjects who recovered following autologous FMT may have done so, at least in part, because of the presence of taxa active in secondary bile acid biosynthesis. We thus hypothesize that members of these groups may similarly play a role in bile acid metabolism, although this suggestion must be interpreted carefully, as this was not mechanistically explored and there remains a paucity of information regarding specific enzymatic activity and the metabolic potential within the *Holdemania* (32).

The effects of antibiotics on the intestinal microbiome have been well documented and include a reduction in alpha diversity and in the relative abundances of taxa within the *Bacteroidetes* and *Firmicutes* phyla and an expansion of *Proteobacteria* (21, 22). Furthermore, several studies have shown that antibiotic-induced changes in the microbiome occur quickly, within days of antimicrobial administration (3, 16, 33). While alpha diversity has been shown to rapidly rebound post-antibiotic exposure, the community composition remains altered, even years later (3, 33). Therefore, it is not surprising that the intestinal microbiomes of autologous FMT recipients failed to rapidly return to those of donor-like assemblages. While they did increase in taxonomic similarity through the 8-week time point, genera that were prominent in donor and heterologous FMT samples, such as *Bacteroides*, failed to recover in autologous FMT subjects. These findings are likely associated with the high recurrence rate associated with antibiotic therapy (34).

In contrast to the increasing focus on defined consortia and augmented donor material for FMT, several studies have examined the success of engraftment of all fecal microbiota as a potential marker for successful recovery (21, 23, 35–38). A majority of studies have assessed taxonomic similarities between recipient and donor samples, interpreting engraftment as the number or percentage of taxa unique to the donor that are found in the patient post-FMT (21, 35, 36). Others have relied on correlations between patient and donor communities based on taxonomic data (23, 38). However, both of these methods are subject to loss of information by binning OTUs into taxa and may be subjected to biases associated with taxonomic databases (37). SourceTracker has previously been used in environmental and clinical studies to effectively determine sources of contamination using a Bayesian approach without taxonomic binning (28, 39, 40). This methodology was employed here to assess donor engraftment as the percentage of patient samples attributable to donor communities. SourceTracker found low similarity to pre-FMT communities in autologous FMT subjects, except immediately prior to recurrence for patient RS02, indicating that the shift in this community failed to show sufficient differentiation from the initial dysbiotic state. Similarly, donor engraftment in heterologous FMT and follow-up FMT samples was high, but incomplete engraftment was observed due to differentiation of patient communities, as has been observed previously (23). The finding that heterologous FMT subjects showed greater percentages of engraftment than follow-up FMT subjects may suggest that the microbiomes of follow-up subjects were more complex due to an initial autologous FMT, or it could indicate a more inhibitory effect of a second follow-up antibiotic regimen prior to follow-up FMT.

High-resolution taxonomic shifts associated with FMT are increasingly being studied in order to augment FMT material and find less-invasive, more-efficient assemblages of bacteria to resolve rCDI, and possibly other conditions. Results of this study indicate that the functional profile of either the pre-FMT or donor material communities should be carefully considered, as functional redundancy may allow for a variety of efficacious consortia (41). In addition, variability in donor microbiota also makes it difficult to assess what communities are needed to restore intestinal health. In the current study, only a limited number of patients who typically received material from a unique donor was used, thus impeding a definition of a more or less efficacious consortium, and further study using a universal donor may help elucidate the most efficacious consortia. Moreover, geographic variability may influence which functionally important taxa are present in donor and patient samples, since notably different clinical outcomes were observed with autologous FMT between New York and Rhode Island samples in the randomized clinical trial, and significant differences in pre-FMT communities were observed between sites (27). Importantly, characterization of the patient’s intestinal microbiome prior to FMT may indicate the presence of taxa that potentially provide natural resistance to rCDI following antibiotic therapy. Despite these apparent difficulties, SourceTracker used in conjunction with FMT provides a promising tool to address the success of engraftment at an OTU level and could potentially be used to identify species efficient at engraftment and/or highly efficacious at resisting infection, in cases of low percentages of engraftment.

## MATERIALS AND METHODS

### Study design.

Complete details of the randomized clinical trial of fecal microbial transplantation are described elsewhere (27). Briefly, a cohort of 24 subjects with rCDI were randomized to receive either colonoscopic autologous fecal microbiota transplantation (FMT) using their own stool (*n* = 14) or colonoscopic heterologous FMT using healthy donor stool (*n* = 10). All samples for FMT were provided on the day of FMT, <6 h prior to the procedure and were refrigerated until use. All subjects were healthy and on a regimen of vancomycin for at least 10 days; the antibiotic was discontinued 2 or 3 days prior to FMT. Patient and donor fecal samples were collected prior to FMT, and patient fecal samples were collected at 2 and 8 weeks post-FMT. Subjects who experienced recurrence received a second course of vancomycin and FMT using open-label healthy donor material. Clinical cure was described as a resolution of diarrhea and no CDI recurrence throughout the 8-week follow-up period without the need for antibiotics.

### Bacterial community characterization.

DNA was extracted from fecal samples, held at −80°C prior to extraction, using the PowerSoil DNA isolation kit (Mo Bio Laboratories, Inc., Carlsbad, CA, USA) as described previously (27). The V5-V6 hypervariable regions of the 16S rRNA gene were amplified and sequenced using the BSF786/1046R bar-coded primer set, and sequence data were processed as previously described using mothur (version 1.34.0) (42, 43). Data are deposited in the Sequence Read Archive of the National Center for Biotechnology under BioProject GenBank accession number SRP066964. Sequence data were trimmed for quality as described previously (43), based on quality scores, homopolymer lengths, the presence of ambiguous bases, and 2% preclustering. Initial sequence alignment was performed against the SILVA database (version 119) (44), and chimeras were identified and removed using UCHIME (45). For comparisons among sample groups, all samples were rarefied to 25,000 sequence reads by random subsampling (46), and samples with fewer sequence reads were removed from the data set. OTUs were assigned at 97% similarity using the furthest-neighbor algorithm, and taxonomic classification was performed against the version 14 database release from the Ribosomal Database Project (47). SourceTracker (28) was used with default parameters to determine the percentage of donor engraftment for heterologous FMT and follow-up FMT subjects and the percentage of the pre-FMT community present in autologous FMT subjects.

### Statistical analyses.

Alpha and beta diversity statistics, Kruskal-Wallis test, and ordination via principal-coordinate analysis were performed using mothur. All other statistics were calculated using XLSTAT (version 2015.01.0; Addinsoft, Belmont, MA). Shannon indices and abundance-based coverage estimates were calculated to evaluate parametric and nonparametric alpha diversity. Bray-Curtis dissimilarity matrices were used for evaluation of beta diversity and ordination (48). The corr.axes command in mothur using the Spearman method was used to determine OTUs significantly affecting axis position. Analysis of similarity (ANOSIM) was used to evaluate differences in beta diversity (49), and analysis of molecular variance (AMOVA) was used to determine the significance of sample clustering on ordination plots (50). Kruskal-Wallis test was used to determine differences in relative abundances of OTUs among sample groups (51). Differences in alpha diversity and relative abundances of taxa were determined using analysis of variance (ANOVA). All statistics were evaluated with α = 0.05.

## SUPPLEMENTAL MATERIAL

FIG S1  Distribution of abundant genera among Rhode Island subject (RS) samples treated by A-FMT. Clinical outcome is shown in parentheses. Download Figure S1, PDF file, 0.2 MB

FIG S2  Distribution of abundant phyla among donor and Rhode Island subject (RS) samples treated initially by H-FMT. All patients received material from a different donor. Download Figure S2, PDF file, 0.3 MB

FIG S3  Percent of donor community, determined by SourceTracker, in H-FMT and F/U-FMT samples. F/U-FMT samples are shown in bold italics. Where data are not shown, sequence data were not available. Download Figure S3, PDF file, 0.2 MB

FIG S4  Principal-coordinate analyses of donor and subject samples collected at 2 weeks (*r*^2^ = 0.54) (A) and 8 weeks post-FMT (*r*^2^ = 0.55) (B). Donor samples are shown in blue, H-FMT samples in green, F/U-FMT samples in purple, and A-FMT samples in orange. Circles indicate groups that separated significantly by AMOVA (*P* < 0.05). Download Figure S4, PDF file, 0.2 MB
