# Supplementary material for: Complete Microbiota Engraftment Is Not Essential for Recovery from Recurrent Clostridium difficile Infection following Fecal Microbiota Transplantation
Source: mBio. 2016 Dec 20;7(6):e01965-16. doi: 10.1128/mBio.01965-16 (PMC5181777; doi:10.1128/mBio.01965-16)
Supplement: FIG S2 — Distribution of abundant phyla among donor and Rhode Island subject (RS) samples treated initially by H-FMT. All patients received material from a different donor. Download [file mbo006163114sf2.pdf]

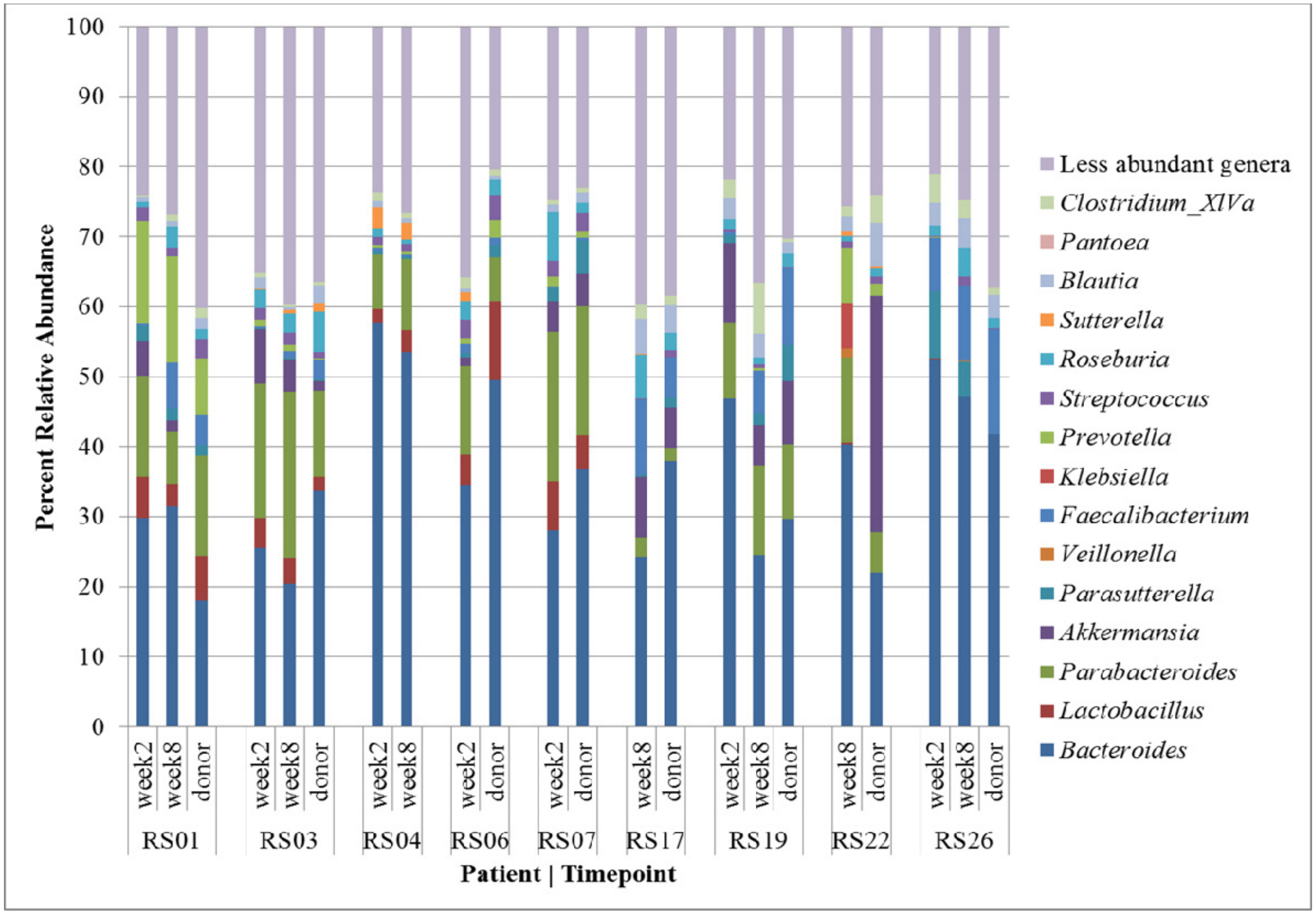

Figure S2. Distribution of abundant phyla among donor and Rhode Island subject (RS) samples treated initially by H-FMT. All patients received material from a different donor
