# Supplementary material for: Complete Microbiota Engraftment Is Not Essential for Recovery from Recurrent Clostridium difficile Infection following Fecal Microbiota Transplantation
Source: mBio. 2016 Dec 20;7(6):e01965-16. doi: 10.1128/mBio.01965-16 (PMC5181777; doi:10.1128/mBio.01965-16)
Supplement: FIG S3 — Percent of donor community, determined by SourceTracker, in H-FMT and F/U-FMT samples. F/U-FMT samples are shown in bold italics. Where data are not shown, sequence data were not available. Download [file mbo006163114sf3.pdf]

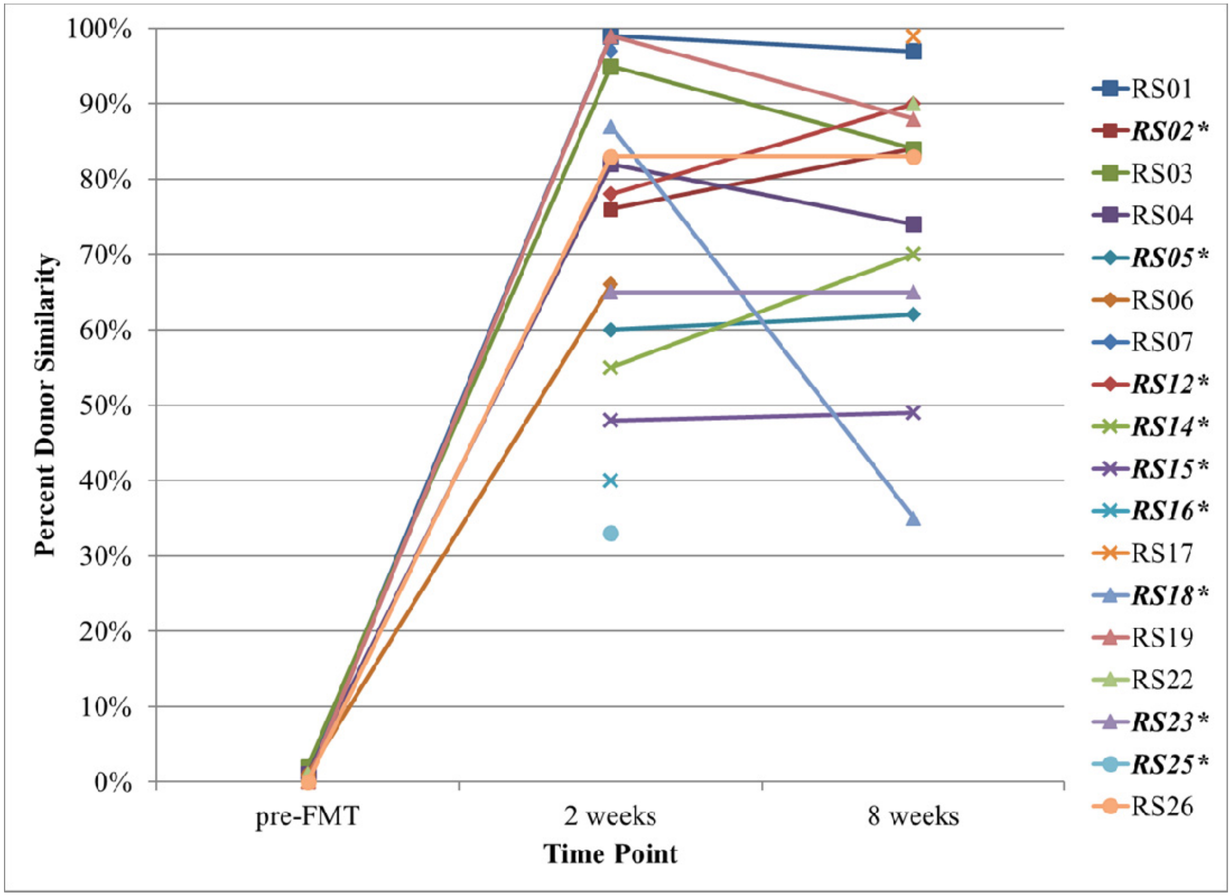

Figure S3. Percent of donor community, determined by SourceTracker, in H-FMT and F/U-FMT samples. F/U-FMT samples are listed in bold italics. Where data is not shown, sequence data was not available.
