# Supplementary material for: Complete Microbiota Engraftment Is Not Essential for Recovery from Recurrent Clostridium difficile Infection following Fecal Microbiota Transplantation
Source: mBio. 2016 Dec 20;7(6):e01965-16. doi: 10.1128/mBio.01965-16 (PMC5181777; doi:10.1128/mBio.01965-16)
Supplement: FIG S4 — Principal-coordinate analyses of donor and subject samples collected at 2 weeks (r2 = 0.54) (A) and 8 weeks post-FMT (r2 = 0.55) (B). Donor samples are shown in blue, H-FMT samples in green, F/U-FMT samples in purple, and A-FMT samples in orange. Circles indicate groups that separated significantly by AMOVA (P < 0.05). Download [file mbo006163114sf4.pdf]

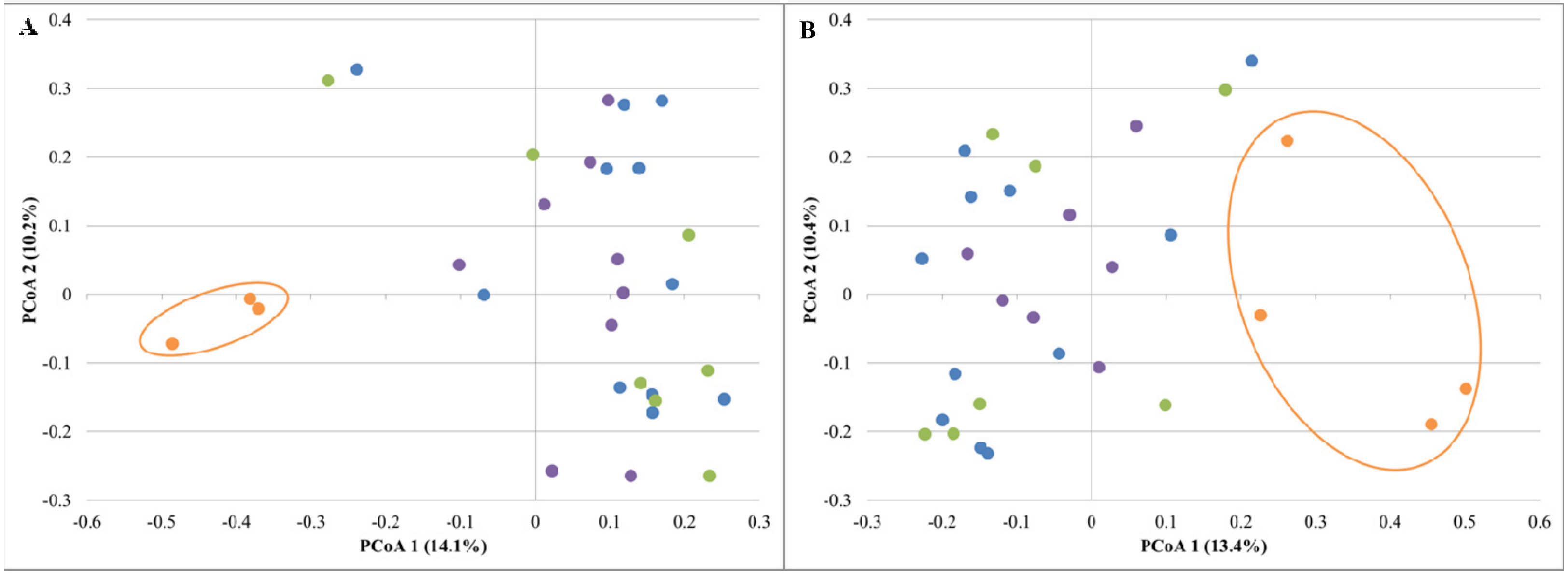

Figure S4 – Principal coordinate analyses of donor and subject samples collected at (A) 2-weeks ($r^2 = 0.54$) and (B) 8-weeks post-FMT ($r^2 = 0.55$). Donor samples are shown in blue, H-FMT samples in green, F/U-FMT samples in purple, and A-FMT samples in orange. Circles indicate groups that separated significantly by AMOVA ($P < 0.05$).
